# Physicochemical Variability and Quality of Honey from the Zagreb Region: The Role of Botanical Origin and Spatial Structure

**DOI:** 10.3390/foods15132311

**Published:** 2026-06-29

**Authors:** Luka Rumora, Ivan Brkić, Nada Vahčić, Ivana Rumora Samarin

**Affiliations:** 1 University of Zagreb Faculty of Geodesy, 10000 Zagreb, Croatia; luka.rumora@geof.unizg.hr (L.R.); ivan.brkic@geof.unizg.hr (I.B.); 2 University of Zagreb Faculty of Food Technology and Biotechnology, 10000 Zagreb, Croatia

**Keywords:** honey quality, physicochemical parameters, urban beekeeping, principal component analysis, spatial autocorrelation, Zagreb region

## Abstract

The physicochemical composition of honey reflects its botanical origin, environmental conditions, and post-harvest handling; however, studies that combine long-term monitoring data with multivariate and spatial analyses at a metropolitan scale remain limited. This study analyzed acacia, chestnut, flower, and meadow honeys from the Zagreb metropolitan region, Croatia, using a long-term physicochemical quality dataset from the Zzzagimed International Honey Quality Competition collected between 2007 and 2019. From a total database of 351 samples, 82 georeferenced samples from the City of Zagreb and Zagreb County were selected and evaluated for moisture, electrical conductivity, free acidity, and hydroxymethylfurfural (HMF). Most samples met general honey quality requirements: only three samples exceeded the moisture threshold of 20%, and no samples exceeded the limits for HMF or free acidity. Electrical conductivity and free acidity provided the clearest differentiation among honey types, ranging from 0.22 to 1.47 mS/cm and from 9.77 to 27.38 meq/kg, respectively. Multivariate analysis showed that physicochemical variability was structured primarily by honey type, with the first two principal components explaining 62.8% of the total variance. Spatial analyses revealed weak to moderate spatial structure, with residual autocorrelation retained only for free acidity. Honeys from the Zagreb region showed good physicochemical quality, and their variability was driven mainly by declared botanical origin rather than by broad City–County differences.

## 1. Introduction

Honey is a naturally sweet substance produced by honey bees (*Apis mellifera*) from the nectar of melliferous plants, secretions of living plant parts, or excretions of plant-sucking insects on living plant parts. Bees collect these materials, transform them by adding specific substances of their own, reduce their water content, and deposit the resulting product in honeycomb cells to ripen. As a natural bee product, honey has a complex chemical composition that reflects its botanical origin, environmental conditions, beekeeping practices, and post-harvest handling. Because of this sensitivity to both floral and environmental factors, physicochemical parameters such as moisture content, electrical conductivity, free acidity, diastase activity, hydroxymethylfurfural (HMF), and proline are widely used as indicators of honey quality, authenticity, maturity, and storage history [1,2,3]. Consumers value honey not only as a natural sweetener, but also for its sensory diversity, perceived naturalness, botanical identity, and association with local and traditional food systems. These characteristics, together with physicochemical quality parameters form the basis of national and European regulatory standards for honey quality assessment [2]. Such monitoring has become increasingly important due to growing concern regarding honey fraud and authenticity in the European market, where routine quality assessment and traceable regional datasets can support transparency, even if they do not directly test for adulteration. In addition to fraud and authenticity, recent honey safety research has increasingly focused on contaminants such as heavy metals, pesticide residues, veterinary drug residues, and persistent emerging pollutants. Although these contaminants were outside the analytical scope of this study, they highlight the broader need for traceable regional honey datasets and integrated monitoring frameworks [4,5,6]. Moisture content relates to honey maturity, stability, and susceptibility to fermentation, while HMF is commonly used as an indicator of overheating, aging, or prolonged storage. Free acidity reflects the organic acid profile and may increase due to fermentation-related changes, whereas electrical conductivity is closely associated with mineral content and is strongly influenced by botanical origin. Unlike general parameters such as moisture, HMF, and free acidity, electrical conductivity must be interpreted with regard to honey type because certain botanical categories, especially honeydew and some unifloral honeys, naturally exhibit higher conductivity values [1,3].

Extensive research has shown that honey composition varies substantially among botanical types. Acacia honeys are typically characterized by low electrical conductivity, relatively low acidity, and light color, whereas chestnut and many polyfloral honeys tend to exhibit higher conductivity and acidity, reflecting greater mineral content and a more complex floral origin [7,8]. Surveys conducted in different geographical regions generally show that most commercial and regional honeys comply with accepted quality limits for moisture, HMF, and free acidity, although occasional deviations may occur due to insufficient ripening, prolonged storage, or inadequate processing conditions [9,10]. Such studies provide an important reference framework for evaluating honey quality and supporting the development of legislative and regulatory frameworks in new regions and production contexts.

In recent years, increasing attention has been given to honey produced in urban and peri-urban environments. Urban beekeeping has expanded in many cities worldwide, raising questions about whether honeys produced in densely built environments differ systematically from those originating in surrounding rural landscapes. Urban ecosystems may expose honey bees to distinct environmental conditions, including altered vegetation composition, microclimatic effects, and potential pollution sources. Nevertheless, previous studies suggest that physicochemical differences between urban and rural honeys are often smaller than differences associated with botanical origin, and that urban honeys frequently remain within typical quality ranges [11,12]. This indicates that urbanization alone may not be sufficient to explain honey physicochemical variability without considering floral origin, local vegetation, and production conditions. Urban beekeeping is also relevant to sustainable urban food systems, as it raises public awareness of pollinator ecosystem services, local food production, and the connections between biodiversity and nutrition. Pollinators contribute to the production of many nutrient-rich foods, including fruits, vegetables, nuts, and seeds [13,14], and are therefore linked to broader sustainability priorities such as food security, sustainable agriculture, and biodiversity. In this context, urban beekeeping is indirectly connected with the Sustainable Development Goals, particularly SDG 2, which promotes food security, improved nutrition, and sustainable agriculture [15]. However, urban beekeeping must be managed responsibly, as hive density, floral resource availability, and interactions with wild pollinators can affect sustainability [16].

Beyond simple comparisons of mean values, multivariate statistical approaches have become increasingly important for analyzing honey composition. Chemometric techniques such as principal component analysis (PCA) and cluster analysis allow simultaneous evaluation of multiple physicochemical variables and can reveal underlying compositional gradients related to botanical or geographical origin. Previous studies have shown that PCA-based approaches can support the discrimination of honey types and identify the physicochemical parameters most responsible for sample differentiation [17,18]. Similar multivariate strategies have also been used to examine honey variability within protected landscapes and geographically defined production areas [19].

Spatial analytical methods offer an additional perspective by linking physicochemical measurements to geographic locations. Geographic information systems (GIS), spatial autocorrelation statistics, and interpolation methods have been used to visualize regional variation in physicochemical parameters and to explore potential spatial gradients in honey quality. For example, Kaur et al. [20] combined physicochemical analysis with GIS techniques to map honey characteristics across large areas of northern India. These approaches show that spatially referenced honey datasets can yield valuable insights into regional variability when interpreted carefully. However, most spatial analyses of honey composition have focused on large geographic areas or protected natural regions, with relatively little attention to finer-scale spatial patterns within metropolitan environments.

Despite extensive research on honey physicochemical properties, most studies use cross-sectional datasets or focus on single analytical approaches. Long-term datasets from routine quality monitoring programs or competition-based evaluations remain underutilized, especially when combined with multivariate and spatial analytical frameworks. Additionally, relatively few studies have examined honey variability within heterogeneous metropolitan environments, where interactions among urban, peri-urban, and rural systems may influence honey composition.

The Zagreb metropolitan region, comprising the City of Zagreb and the surrounding Zagreb County, serves as a relevant model for studying honey physicochemical variability in a heterogeneous metropolitan context. Within a relatively compact area, the region encompasses a dense urban core, suburban settlements, agricultural landscapes, riparian habitats, meadow vegetation, and the forested slopes of Medvednica Mountain. This spatial heterogeneity creates a diverse mosaic of nectar sources and broad environmental context, while the Zzzagimed International Honey Quality Competition provides a long-term dataset of honey samples analyzed for physicochemical quality under standardized laboratory conditions. Thus, Zagreb offers an opportunity to examine whether physicochemical variability is more strongly associated with declared botanical origin, broad administrative zone, or residual spatial structure.

The available metadata do not include direct measurements of land use, pollution exposure, apiary management, or microclimatic conditions. Therefore, the study was designed as an exploratory analysis of physicochemical variability rather than a direct test of urbanisation or environmental exposure effects. Because the available metadata do not include direct measures of land use, pollution exposure, vegetation composition, apiary management, or microclimate, the City–County comparison was not used as a direct urbanisation gradient. Instead, it was treated as a coarse administrative proxy for broad spatial context.

This study focuses on acacia, chestnut, meadow, and mixed flower honeys from the Zagreb region. Rather than treating the City–County division as a direct measure of urbanization, this classification is used as a coarse administrative proxy for broad spatial context and is interpreted with caution. By combining descriptive statistics, quality compliance screening, multivariate analysis, temporal assessment, and GIS-based spatial methods, the study evaluates whether physicochemical variability is primarily associated with declared botanical origin, administrative zone, or residual spatial structure.

The main scientific gap addressed by this study is the limited use of long-term competition-based honey quality datasets for integrated physicochemical, multivariate, and spatial analysis in metropolitan regions. We hypothesised that declared botanical origin would explain more physicochemical variability than the broader City–County administrative classification, while spatial analysis could reveal whether residual geographic structure remained after accounting for honey type.

Specifically, the aims of this study were to:

(i) Characterize the physicochemical properties and quality compliance of acacia, chestnut, meadow, and mixed flower honeys from the Zagreb region;
(ii) Evaluate whether honey samples from the City of Zagreb and Zagreb County differ in selected physicochemical parameters, using the City–County classification as a coarse administrative proxy;
(iii) Examine multivariate patterns in honey composition using principal component analysis and k-means clustering;
(iv) Assess temporal variability in moisture and HMF over the 2007–2019 period;
(v) Explore spatial autocorrelation, residual spatial structure, and spatial visualization of selected physicochemical parameters using GIS-based methods.

By integrating physicochemical measurements with multivariate and spatial analyses, this study provides a comprehensive assessment of honey quality in the Zagreb metropolitan region and contributes to a better understanding of the relative importance of declared botanical origin, administrative zoning, and local spatial structure in shaping honey physicochemical variability.

## 2. Materials and Methods

### 2.1. Study Area, Honey Samples and Georeferencing

**Figure 1:**
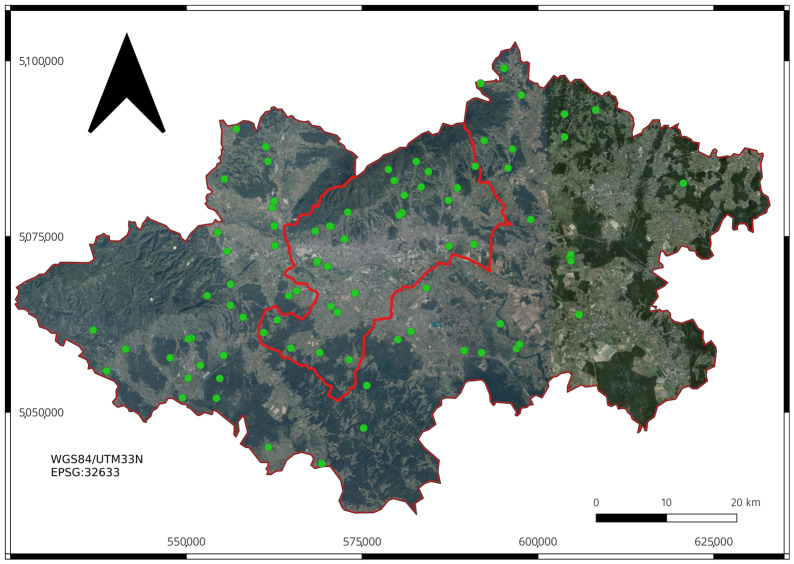
Study area and spatial distribution of honey sampling locations in the City of Zagreb and Zagreb County. Red lines indicate administrative boundaries, and green dots indicate honey sampling locations.

The study was conducted in the City of Zagreb and Zagreb County in northwestern Croatia (Figure 1). Together, these administrative units form a heterogeneous metropolitan landscape that includes a densely built urban core, suburban settlements, agricultural areas, forested slopes, riparian zones, and meadow habitats. The region has a temperate continental climate, with warm summers and moderately cold winters, influenced by the Sava River valley and the forested slopes of Medvednica Mountain, which create local variation in temperature, humidity, and vegetation [21,22].

From a beekeeping perspective, the area is characterized by a mosaic of nectar sources, including acacia (*Robinia pseudoacacia*) in lowland and riparian zones, chestnut (*Castanea sativa*) forests on the slopes of Medvednica, urban and suburban ornamental flora, and meadow and pasture habitats in the wider county. These heterogeneous environmental conditions provide an appropriate setting for examining how declared botanical origin and local geography relate to the physicochemical properties of honey [23,24,25].

The data used in this study were obtained from the honey quality evaluation system established within the Zzzagimed International Honey Quality Competition. The dataset includes analytical results of honey samples collected between 2007 and 2019, representing a form of long-term monitoring of physicochemical honey quality. Samples were collected as part of regular competition activities, and included physicochemical and sensory analyses parameters in accordance with the applicable evaluation protocols. Within the Zzzagimed competition, beekeepers submit honey exclusively from their own production, and submitted samples must not be older than one year at the time of submission. The database did not systematically record whether samples were submitted in the Croatian national honey jar; therefore, jar type was not included as an analytical variable. All submitted samples are analyzed in the laboratory using standardized and validated analytical methods in accordance with national and European Union quality requirements. Analytical results are stored in a central database together with metadata, including production year, declared botanical origin, and producer location.

From 2007 to 2019, the Zzzagimed database contained 351 honey samples from different parts of Croatia. Because this study focused on spatial and multivariate analysis within the Zagreb metropolitan region, only samples with available geographical coordinates and located within the administrative boundaries of the City of Zagreb or Zagreb County were retained. This resulted in a georeferenced Zagreb-region subset of 82 samples. Although this subset represents a reduced portion of the full monitoring database, it includes the main honey types recorded in the study region and covers the full study period. Because samples were submitted voluntarily to a honey competition, the dataset should not be interpreted as a probabilistic sample of all honey produced in the Zagreb region. It may overrepresent beekeepers who participate in quality evaluation activities and whose products are prepared for competition submission. Therefore, the dataset was treated as a long-term, quality-related monitoring dataset suitable for exploratory analysis.

Four dominant honey types were examined: acacia honey (*Robinia pseudoacacia*; “Bagrem”), chestnut honey (*Castanea sativa*; “Kesten”), meadow polyfloral honey (“Livada”), and mixed flower honey (“Cvjetni”). Botanical origin was based on beekeeper declaration and the standard competition evaluation procedures. Pollen analysis was not performed for all samples; therefore, botanical categories are interpreted as declared and competition-assessed honey types rather than independently confirmed botanical classes. The database did not systematically include protected geographical indication or protected designation of origin information; therefore, geographical quality schemes were not used as analytical grouping factors.

Geographical coordinates were available for all 82 samples in the Zagreb-region subset. Coordinates were recorded at sampling or derived from producer addresses using official geocoding sources. Point locations were imported into an sf-based spatial database in R version 4.5.2 and transformed to the WGS 84/UTM zone 33N coordinate reference system (EPSG:32633) for spatial analyses. Administrative boundaries for the City of Zagreb and Zagreb County were obtained from GIS boundary datasets and transformed to the same coordinate reference system. Samples were spatially joined to these polygons and assigned to one of two administrative zones: City of Zagreb or Zagreb County.

The City–County classification was used as a coarse administrative proxy for urban versus peri-urban/rural conditions. Because administrative boundaries do not fully represent land-use intensity, vegetation composition, pollution exposure, or the immediate surroundings of apiaries, this classification was interpreted cautiously and was not treated as a direct quantitative measure of urbanization. Samples with incomplete information for individual physicochemical parameters were excluded from analyses requiring those parameters but retained where possible for descriptive or variable-specific analyses. The spatial dataset was checked for missing values and duplicate coordinates before spatial analysis.

### 2.2. Physicochemical Analyses

All physicochemical analyses were conducted using standardized and validated methods recommended by the International Honey Commission (IHC) and relevant European regulations [3]. Honey samples were collected directly from beekeepers. For each sample, two parallel aliquots were prepared in identical 370 mL glass containers, filled and hermetically sealed by the beekeepers. All jars were anonymised with coded identifiers and contained no beekeeper information, geographical indication marks, or quality labels. Consequently, analysts were blinded to the commercial status of the samples, including whether the honey was marketed within the national honey jar system or associated with any geographical indication or quality scheme. Samples were stored at room temperature and protected from direct light before and during analytical procedures.

Moisture content (% m/m) was determined by refractometry at 20 °C using a refractometer (Model I, Carl Zeiss, Jena, Germany) in accordance with AOAC International Official Methods. Electrical conductivity (mS cm^−1^) was measured in a 20% (*w*/*v*) honey solution with a calibrated conductivity meter (Mettler-Toledo 8603, Mettler-Toledo GmbH, Schwerzenbach, Switzerland). Free acidity (meq kg^−1^) was determined by titration with 0.1 mol L^−1^ NaOH to pH 8.3 using a pH meter (Mettler-Toledo S220, Mettler-Toledo GmbH, Schwerzenbach, Switzerland). Diastase activity was assessed using the Phadebas method. Hydroxymethylfurfural (HMF, mg kg^−1^) was determined by high-performance liquid chromatography with UV/VIS diode-array detection at 285 nm, using a Luna 5u C18 column (100 Å, 250 × 4.6 mm; Phenomenex, Torrance, CA, USA). The mobile phase consisted of degassed water:methanol (90:10, *v*/*v*), and the flow rate was 1.0 mL min^−1^. Proline content (mg kg^−1^) was determined spectrophotometrically based on its reaction with ninhydrin using a UV-1280 spectrophotometer (Shimadzu Corporation, Kyoto, Japan). All measurements were performed in duplicate according to laboratory quality assurance procedures.

Additional physicochemical parameters, including total reducing sugars, sucrose content, ash content, invertase activity, and optical rotation, were determined according to AOAC and IHC methods as part of the broader quality assessment but were not included in the present statistical analyses due to incomplete data.

Although additional quality and sensory parameters were available in the broader competition evaluation system, the present statistical and spatial analyses focused on four variables with the most complete and comparable datasets across the georeferenced samples: moisture, electrical conductivity, free acidity, and HMF. Diastase activity and proline were measured as part of the broader quality assessment but were not included in the main spatial and multivariate analyses due to lower completeness across the georeferenced subset.

### 2.3. Statistical Analysis and Quality Compliance Assessment

Descriptive statistics, including mean, standard deviation, minimum, and maximum, were calculated for each physicochemical parameter by honey type. The number of samples was also summarized by honey type and administrative zone to assess dataset balance, following general recommendations for the analysis of food composition datasets [26].

City–County comparisons were conducted as secondary exploratory analyses. Moisture and electrical conductivity were compared between City and County samples within each honey type using Student’s *t* test when assumptions of normality and homogeneity of variance were met. When these assumptions were not met, Welch’s *t* test or the Mann–Whitney U test was used as appropriate. Normality was assessed using the Shapiro–Wilk test and visual inspection of Q–Q plots, while homogeneity of variance was assessed using Levene’s test. Because multiple comparisons were performed, *p*-values were adjusted using the Benjamini–Hochberg false discovery rate procedure. Given the modest and unbalanced sample sizes, these City–County comparisons were interpreted as exploratory rather than confirmatory evidence of spatial-zone differences [26].

To evaluate the relative contributions of declared honey type and administrative zone more directly, linear models were fitted for each main physicochemical parameter using the structure:

*parameter ~ honey type + administrative zone*

For HMF, a log-transformed response, log(HMF + 1), was used due to its right-skewed distribution and the presence of low or zero values. Type II analysis of variance was used to evaluate the effects of honey type and administrative zone, and adjusted R^2^ values summarized model fit. These models assessed whether administrative zone explained additional variability after accounting for honey type [26,27].

Quality compliance was summarized for general regulatory thresholds for moisture, HMF, and free acidity, based on International Honey Commission methods and applicable national and European honey quality requirements [2,3,28]. Electrical conductivity was not summarized as a single compliance category because conductivity limits depend on honey type. Patterns of missing data were summarised by honey type and parameter (Table A10). As parameter availability varied among variables, descriptive statistics were reported with parameter-specific sample sizes, and analyses requiring complete data, such as principal component analysis, were limited to complete cases.

### 2.4. Multivariate and Temporal Analyses

Principal component analysis (PCA) was conducted to assess the joint variability of the four main physicochemical variables: moisture, electrical conductivity, free acidity, and HMF, following chemometric methods previously used in honey classification and physicochemical profiling studies [17,18,29]. Only samples with complete measurements for all four variables were included in the PCA. After filtering for complete cases, the PCA dataset included 52 samples: Acacia (*n* = 20), Chestnut (*n* = 11), Flower (*n* = 12), and Meadow (*n* = 9). This sample size was considered sufficient for exploratory PCA, as the number of observations substantially exceeded the number of variables analyzed.

Before PCA, all variables were standardized to a mean of zero and unit variance, as is standard when physicochemical variables are measured in different units [17,18,29]. Scores for the first principal components were visualized in scatter plots colored by honey type, and loadings were used to interpret the contribution of individual variables. The first three principal components, which together explained 83.7% of the total variance, were retained for clustering.

K-means clustering was applied to the PCA scores for PC1–PC3 as an exploratory descriptive analysis of sample grouping, consistent with previous multivariate approaches used for honey differentiation [18,29]. Internal clustering diagnostics were examined but did not identify a uniquely optimal number of clusters. Therefore, cluster solutions for k = 2–6 were also evaluated against biologically expected macro-groups defined as acacia, chestnut, and polyfloral honeys, with flower and meadow honeys combined into a single polyfloral category. Agreement between clusters and macro-groups was assessed using the adjusted Rand index, normalised mutual information, purity, and macro-F1. The k = 3 solution was retained as an exploratory and biologically interpretable solution because it showed the strongest agreement with the expected macro-groups and provided a parsimonious summary of the main physicochemical groupings (Table A11). Cluster stability was improved by using 50 random starts, and cluster composition was summarised by honey type.

Temporal variability was examined by aggregating observations by production year and honey type. Moisture and HMF were selected for temporal analysis because these variables had the most consistent coverage across the analyzed period and are commonly used as key indicators of honey quality and storage-related changes [1,3]. For each year–type combination, mean values were calculated only when at least three samples were available. Annual means were plotted to visualize inter-annual variation in moisture and HMF from 2007 to 2019.

Simple linear regression models were fitted to annual mean values to assess broad temporal trends. Because the dataset was unbalanced across years and honey types, and the number of observations per year–type combination was limited, temporal analyses were interpreted as exploratory rather than confirmatory evidence of long-term trends [26].

### 2.5. Spatial Analysis, Interpolation, and Local Clustering

Spatial autocorrelation was assessed using Global Moran’s I, which evaluates whether geographically neighboring observations tend to have similar values [30]. Moran’s I values range from −1, indicating dispersion, to +1, indicating clustering, with values near zero indicating spatial randomness. Spatial weights were defined using a k-nearest neighbor approach with k = 4. This approach ensures that each observation has the same number of neighbors and is suitable for datasets with uneven spatial sampling density [31].

Global Moran’s I was first calculated for the observed values of moisture, electrical conductivity, HMF, and free acidity. To determine whether observed spatial structure remained after accounting for honey type, residual Moran’s I was then calculated using residuals from linear models of the form

*parameter ~ honey type*

This residual spatial autocorrelation analysis distinguished spatial structure associated with the geographic distribution of honey types from residual geographic variation within physicochemical parameters. The same k-nearest neighbor spatial weights were used for both raw and residual Moran’s I analyses.

Spatial interpolation was used to provide exploratory visualizations of spatial variation in selected physicochemical parameters across the Zagreb region. Moisture and free acidity were selected because they showed evidence of spatial structure in the global spatial analysis, while free acidity also retained significant residual spatial autocorrelation after accounting for honey type.

Inverse distance weighting (IDW) was applied as a deterministic interpolation method commonly used for visualizing spatial gradients when sampling density is limited or unevenly distributed [32,33,34]. IDW estimates values at unsampled locations as weighted averages of neighboring observations, with weights decreasing as distance increases. Interpolation was performed in the WGS 84/UTM zone 33N coordinate reference system using all available samples for each analyzed variable. The IDW power parameter was selected by leave-one-out cross-validation, testing powers between 1 and 3 and choosing the value that minimized the root mean square error (RMSE).

Ordinary kriging was also evaluated as a methodological benchmark using spherical, exponential, Gaussian, and Matérn variogram models [32,33,35]. Because spatial autocorrelation was weak to moderate and the sampling network was relatively sparse, kriging was interpreted as a comparative geostatistical benchmark rather than the primary predictive interpolation method. Model performance for IDW and ordinary kriging was evaluated using leave-one-out cross-validation, with RMSE, mean absolute error, and coefficient of determination (R^2^) reported as performance metrics. The resulting maps were interpreted as exploratory visualizations of relative spatial patterns, not as precise predictions at unsampled locations.

Local spatial structure was examined using Local Indicators of Spatial Association (LISA) based on the local Moran statistic [36]. LISA analysis was applied to free acidity because this parameter showed the strongest global spatial autocorrelation and retained significant residual spatial autocorrelation after accounting for honey type. LISA decomposes global spatial autocorrelation into local components and identifies locations contributing to spatial clustering or spatial outliers.

Each sample was classified into one of four local spatial association categories: high–high clusters, low–low clusters, high–low outliers, and low–high outliers. Samples without statistically significant local spatial association were classified as not significant. Local Moran statistics were evaluated using permutation testing with 999 random permutations and α = 0.05. Because multiple local tests were performed and the number of significant locations was small, LISA results were interpreted as exploratory indicators of localized spatial structure rather than definitive evidence of widespread clustering.

All statistical and spatial analyses were conducted in R version 4.5.2 [27]. Spatial data were processed with the sf package (version 1.0.20); spatial autocorrelation analyses used spdep (version 1.3.11); interpolation used gstat (version 2.0.7); and visualization used ggplot2 (version 3.5.2). Additional data manipulation and statistical summaries were performed with the dplyr (version 1.1.4), tidyr (version 1.3.1), broom (version 1.0.8), and car (version 3.1.3) packages.

## 3. Results

### 3.1. Composition and Quality Profile of Honey Samples in the Zagreb Region

A subset of 82 georeferenced honey samples from the City of Zagreb and Zagreb County was used for descriptive and spatial analyses. This subset was extracted from the full Zzzagimed database, which contained 351 honey samples collected between 2007 and 2019. The analyzed Zagreb-region subset included four main honey types: acacia honey (*n* = 30), flower honey (*n* = 19), chestnut honey (*n* = 18), and meadow honey (*n* = 15). The distribution of samples by honey type and administrative zone is provided in Table A7, and the sample selection flow is summarized in Table A6.

Clear differences in physicochemical composition were observed between the honey types (Table 1).

**Figure 2:**
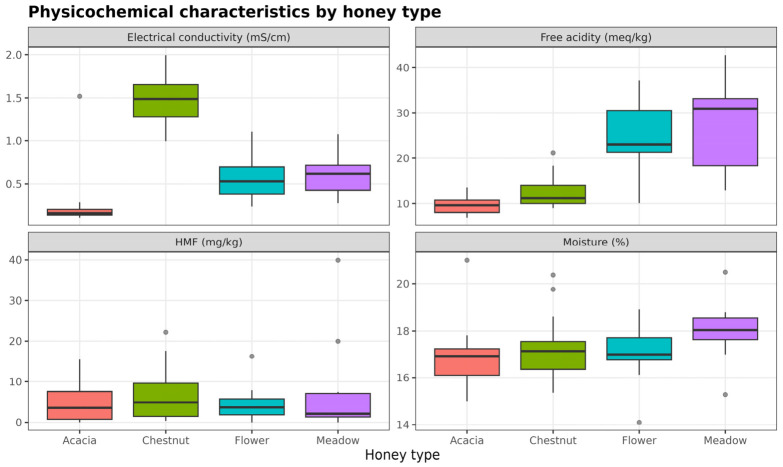
Distribution of physicochemical parameters across honey types.

Clear differences in physicochemical composition were observed among honey types (Table 1; Figure 2). Mean moisture values varied within a relatively narrow range, from 16.78% in acacia honey to 18.00% in meadow honey. In contrast, electrical conductivity and free acidity showed much stronger differentiation among honey types.

Acacia honey had the lowest mean electrical conductivity (0.22 mS/cm), while chestnut honey had the highest mean value (1.47 mS/cm). Flower and meadow honeys had intermediate conductivity values, with means of 0.57 and 0.60 mS/cm, respectively. The relatively high standard deviation of electrical conductivity in acacia honey was influenced by one sample with an unusually high value of 1.52 mS/cm. Because no analytical error could be confirmed, this value was retained in the dataset. However, its presence should be considered when interpreting acacia honey variability, as it may reflect mixed floral contribution, local environmental variability, or uncertainty in declared botanical origin. A sensitivity summary is provided in Table A8.

Free acidity also differed substantially among honey types. Acacia honey had the lowest mean free acidity (9.77 meq/kg), followed by chestnut honey (12.91 meq/kg). Flower and meadow honeys had higher mean acidity values, 24.54 and 27.38 meq/kg, respectively. HMF concentrations were generally low across all honey types, with mean values ranging from 4.29 mg/kg in flower honey to 7.01 mg/kg in meadow honey. Together with the relatively narrow range of moisture content, these low HMF values indicate no evidence of widespread overheating or prolonged storage effects in the analysed samples. However, actual storage conditions before sample submission were not measured, and this interpretation should therefore be considered indirect.

Boxplots display moisture, electrical conductivity, free acidity, and HMF content for acacia, chestnut, flower, and meadow honeys.

Quality compliance screening showed that most samples were within general honey quality limits. Only three of 82 samples exceeded the general moisture threshold of 20%, corresponding to 3.7% of the analyzed samples. No samples exceeded the general limits for HMF (0/72) or free acidity (0/62). These results are summarized in Table A5. Overall, the descriptive statistics indicate that Zagreb-region honeys were generally compliant with quality requirements, while electrical conductivity and free acidity provided the clearest differentiation among honey types.

### 3.2. Effects of Honey Type and Administrative Zone

Differences in physicochemical parameters between samples from the City of Zagreb and Zagreb County were evaluated as secondary exploratory analyses within each honey type for moisture and electrical conductivity. Overall, differences between the two administrative zones were modest and not consistent across honey types. After Benjamini–Hochberg false discovery rate correction, none of the City–County comparisons remained statistically significant (Table A4). Flower honey showed an unadjusted difference in moisture between zones (*p* = 0.012), but this effect was not statistically robust after correction for multiple testing (adjusted *p* = 0.095). These findings suggest that broad administrative zoning did not produce consistent differences in the analyzed physicochemical parameters.

To further evaluate the relative importance of botanical origin and administrative zone, linear models were fitted for each main physicochemical parameter using honey type and administrative zone as explanatory variables.

Type II analysis of variance was used. HMF was analyzed using log(HMF + 1). Adjusted R^2^ values summarize model fit.

The linear models showed that honey type was a significant predictor of moisture, electrical conductivity, and free acidity, whereas administrative zone was not significant for any analyzed parameter (Table 2). Honey type had a significant effect on moisture (*p* = 0.013), electrical conductivity (*p* < 0.001), and free acidity (*p* < 0.001). The strongest model fit was observed for electrical conductivity, with an adjusted R^2^ of 0.766, and for free acidity, with an adjusted R^2^ of 0.632, indicating that a substantial proportion of variability in these parameters is explained by differences among honey types. In contrast, the model for HMF showed no significant effect of honey type or administrative zone and had no explanatory power, suggesting that HMF variability is influenced by factors not captured in the present model.

Administrative zones were not significant for moisture (*p* = 0.652), electrical conductivity (*p* = 0.233), free acidity (*p* = 0.621), or HMF (*p* = 0.856). These results confirm that declared honey type explained substantially more physicochemical variability than the broad City–County classification.

### 3.3. Multivariate Structure of Physicochemical Parameters

**Figure 3:**
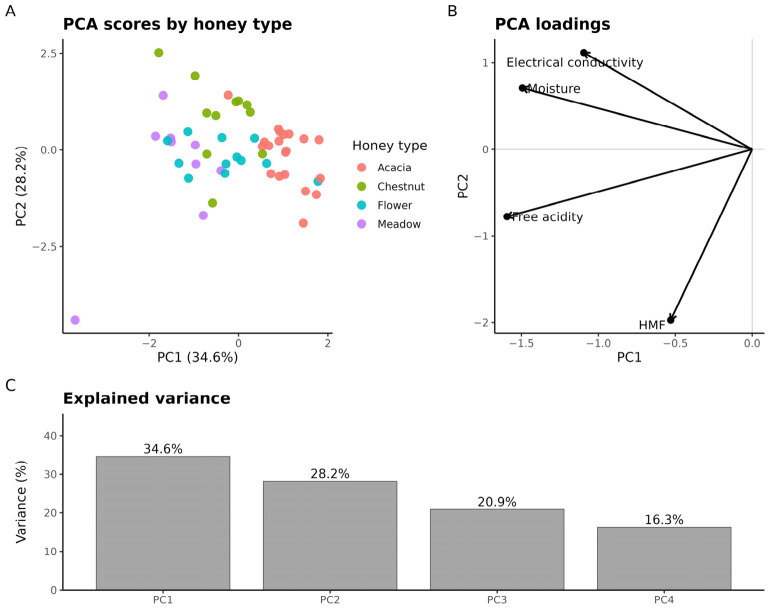
Principal component analysis (PCA) of honey samples from the Zagreb region: (**A**) PCA scores plot showing the distribution of samples by honey type; (**B**) PCA loadings showing the contribution of moisture, electrical conductivity, free acidity, and HMF to the first two principal components; (**C**) percentage of variance explained by the principal components.

Principal component analysis was performed on the complete-case dataset containing moisture, electrical conductivity, free acidity, and HMF measurements (Figure 3). After complete-case filtering, the PCA dataset included 52 samples with complete data for all four variables: Acacia *n* = 20, Chestnut *n* = 11, Flower *n* = 12, and Meadow *n* = 9.

The first two principal components explained 62.8% of the total variance, with PC1 accounting for 34.6% and PC2 for 28.2%. The first three components together explained 83.7% of the total variance (Table A2). The PCA scores showed a clear tendency for samples to group by honey type. Acacia honeys were generally separated from chestnut and polyfloral honeys, while flower and meadow honeys showed partial overlap, reflecting their broader compositional variability.

The PCA loadings indicated that PC1 was mainly associated with free acidity and moisture, with an additional contribution from electrical conductivity. PC2 was most strongly associated with HMF, while electrical conductivity contributed strongly to PC3 (Table A1). Therefore, the separation of honey types in the PC1–PC2 scores plot should be interpreted as a combined physicochemical gradient, rather than as a pattern based solely on conductivity. Chestnut honeys were compositionally distinct in the full multivariate space, mainly due to the strong contribution of conductivity to PC3, while acacia honeys were characterised by low conductivity and low acidity. Flower and meadow honeys occupied intermediate and partially overlapping positions, consistent with their polyfloral origin.

K-means clustering was used as an exploratory descriptive analysis of PCA-based sample grouping. Internal clustering diagnostics did not indicate a uniquely optimal number of clusters. However, external validation against biologically expected macro-groups showed that the k = 3 solution had the highest adjusted Rand index (0.571) and normalised mutual information (0.611). Purity and macro-F1 were also high for k = 3 (0.846 and 0.848, respectively), and equal to the k = 4 solution (Table A11). Therefore, the k = 3 solution was retained as a parsimonious and biologically interpretable exploratory grouping rather than as a definitive classification model.

In the k = 3 solution, one cluster was dominated by acacia honey, one consisted mainly of polyfloral honeys with one chestnut sample, and one was dominated by chestnut honey (Table A3). This pattern was consistent with the PCA results and supports the interpretation that declared honey type was an important source of multivariate structure, although the primary multivariate evidence comes from the PCA scores and loadings.

### 3.4. Temporal Patterns of Moisture and HMF

**Figure 4:**
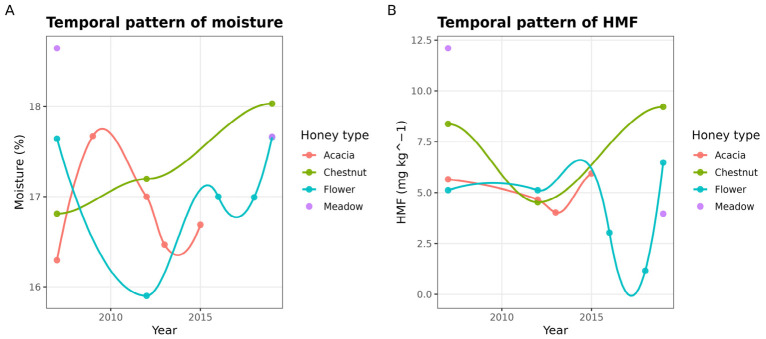
Temporal trends in honey physicochemical parameters in the Zagreb region from 2007 to 2019. (**A**) Annual mean moisture content by honey type; (**B**) annual mean HMF concentration by honey type. Only year–type combinations with at least three samples are shown.

Temporal variability in honey composition was examined using year–type combinations with at least three samples (Figure 4). This filtering resulted in 15 year–type combinations available for temporal analysis. Moisture and HMF were selected because they had the most consistent temporal coverage across the analyzed period.

Annual mean moisture values generally remained within a narrow range across the analyzed period. Among year–type combinations with sufficient replication, mean moisture ranged from 15.90% to 18.65%. Acacia honey showed relatively stable moisture values across available years, while flower, chestnut, and meadow honeys showed moderate interannual variation.

Annual mean HMF concentrations were generally low. Across year–type combinations with sufficient replication and available HMF data, mean HMF values ranged from 1.17 to 12.10 mg kg^−1^, remaining well below the general limit of 40 mg kg^−1^. Occasional higher annual means were observed, particularly in meadow honey in 2007 and chestnut honey in 2019, but these values did not indicate a persistent increase over time.

Exploratory linear regressions of annual mean values did not indicate statistically significant temporal trends for moisture or HMF in honey types with at least three available year–type observations. For moisture, no significant trend was detected for acacia, chestnut, or flower honey, while meadow honey had too few year–type observations for regression-based interpretation. For HMF, no significant trend was detected for acacia, chestnut, or flower honey, while meadow honey again had insufficient replicated time points. Overall, the temporal analysis suggests moderate interannual variation but no clear evidence of systematic long-term deterioration in moisture or HMF during the 2007–2019 period.

### 3.5. Spatial Autocorrelation and Exploratory Interpolation

Spatial autocorrelation was evaluated using Global Moran’s I for the observed physicochemical values and residual Moran’s I after accounting for honey type (Table 3).

Residual Moran’s I was calculated from the residuals of models that included honey type as an explanatory variable.

For the observed values, moisture showed weak but statistically significant positive spatial autocorrelation (Moran’s I = 0.120, *p* = 0.041). Free acidity showed stronger spatial autocorrelation (Moran’s I = 0.209, *p* = 0.002). Electrical conductivity (Moran’s I = 0.050, *p* = 0.281) and HMF (Moran’s I = −0.010, *p* = 0.512) did not show significant global spatial autocorrelation.

After accounting for honey type, residual spatial autocorrelation was no longer evident for moisture, electrical conductivity, or HMF. Residual Moran’s I values were 0.010 for moisture (*p* = 0.3762), −0.059 for electrical conductivity (*p* = 0.7363), and −0.058 for HMF (*p* = 0.7372). In contrast, free acidity retained significant residual spatial autocorrelation after accounting for honey type (residual Moran’s I = 0.266, *p* = 0.0001). This indicates that the spatial pattern of free acidity was not fully explained by the geographic distribution of honey types alone. Because no direct measurements of vegetation composition, land use, road proximity, contaminant levels, apiary management, or microclimate were available, this residual spatial structure should be interpreted as evidence of unexplained local spatial heterogeneity rather than proof of a specific environmental driver.

**Figure 5:**
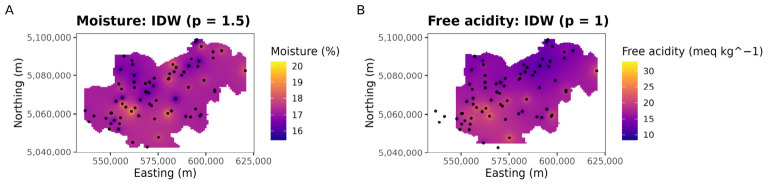
IDW interpolation surfaces for selected physicochemical parameters. (**A**) Moisture; (**B**) free acidity. Surfaces are presented as exploratory visualizations of relative spatial patterns and not as precise predictions at unsampled locations.

Spatial interpolation was then used as an exploratory visualization of moisture and free acidity across the study area (Figure 5). Model performance was evaluated using leave-one-out cross-validation (Table 4).

For moisture, the best-performing IDW model used a power parameter of 1.5 and achieved RMSE = 1.165, MAE = 0.842, and R^2^ = 0.326. The best-performing ordinary kriging model used a Matérn variogram but showed weaker predictive performance, with RMSE = 1.550, MAE = 0.941, and R^2^ = 0.013.

For free acidity, the best-performing IDW model used a power parameter of 1.0 and achieved RMSE = 8.961, MAE = 7.223, and R^2^ = 0.061. The best-performing ordinary kriging model used a Gaussian variogram, with RMSE = 9.660, MAE = 7.943, and R^2^ = 0.001. For both variables, IDW produced lower prediction errors than ordinary kriging. The very low R^2^ values of the kriging models indicate that variogram-based spatial prediction captured only a limited proportion of the observed variability. Therefore, the interpolation maps were interpreted as exploratory visualizations rather than robust predictive surfaces.

### 3.6. Local Spatial Patterns of Free Acidity

**Figure 6:**
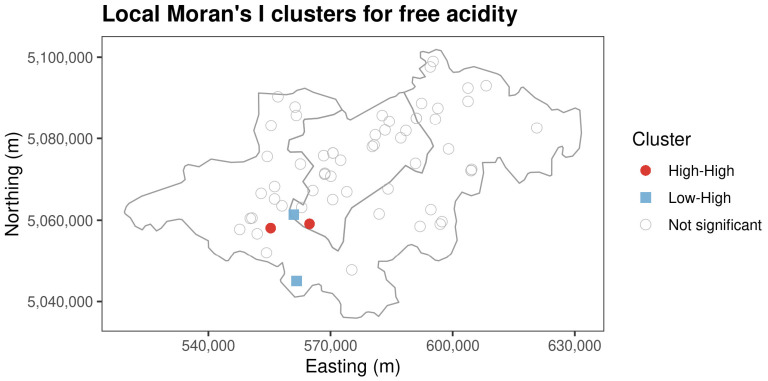
Local Moran’s I cluster map for free acidity in the Zagreb region.

Because free acidity showed significant global and residual spatial autocorrelation, local spatial structure was further examined using Local Indicators of Spatial Association based on the local Moran statistic (Figure 6).

The map shows statistically significant local spatial associations and non-significant locations within the City of Zagreb and Zagreb County.

The LISA analysis was performed on the subset of samples with complete free acidity measurements (*n* = 62). Two high–high clusters were identified, indicating locations where samples with relatively high free acidity were surrounded by neighboring samples with similarly high acidity values. Two low–high spatial outliers were also detected, representing samples with lower acidity values located within areas characterized by higher surrounding acidity.

Most samples did not show statistically significant local spatial association. Specifically, 58 of 62 samples were classified as not significant, while only four samples were assigned to significant LISA categories. No low–low clusters or high–low outliers were detected. These results indicate that local spatial structure in free acidity was present but limited in extent (Table A9). Therefore, the LISA results should be interpreted as exploratory indicators of localized spatial heterogeneity rather than as evidence of widespread spatial clustering.

## 4. Discussion

This study evaluated the physicochemical variability and quality of honey from the Zagreb metropolitan region using a long-term dataset from a quality monitoring and competition-based evaluation system. Overall, the results indicate that the honey samples were largely compliant with general quality requirements and exhibited physicochemical characteristics consistent with their declared botanical origin.

### 4.1. Physicochemical Quality and Botanical Differentiation of Zagreb-Region Honeys

The physicochemical profile of honeys from the Zagreb region indicates generally good product quality and broad compliance with national and European honey quality requirements. Moisture content, HMF, and free acidity were within the expected ranges for most samples. Only three of 82 samples exceeded the general moisture threshold of 20%, and no samples exceeded the general limits for HMF or free acidity. These results suggest that insufficient ripening, excessive water content, overheating, or prolonged storage were not widespread quality problems in the analyzed dataset.

Mean moisture values ranged from 16.78% in acacia honey to 18.00% in meadow honey, consistent with values commonly reported for good-quality honeys [1,9,10]. HMF concentrations were also low, with mean values between 4.29 and 7.01 mg kg^−1^ depending on honey type. Since HMF is widely used as an indicator of heating, aging, and prolonged storage, these values are consistent with the absence of widespread overheating or prolonged storage effects in the analysed samples [1,37]. However, storage conditions before sample submission were not measured directly, so this interpretation should be considered indirect. This is also consistent with the Zzzagimed requirement that submitted honey must not be older than one year at the time of submission.

The largest differences among honey types were observed for electrical conductivity and free acidity, indicating that these parameters provide the strongest differentiation between botanical origins. Acacia honey had the lowest mean electrical conductivity and free acidity, while chestnut honey showed the highest conductivity. Flower and meadow honeys showed intermediate conductivity but higher free acidity. This pattern is consistent with the known physicochemical characteristics of these honey types: acacia honey is typically light, mild, and low in minerals, while chestnut and many polyfloral honeys are richer in minerals and organic acids [7,8]. Similar differentiation of Croatian honeys by physicochemical parameters has also been reported previously, where acacia, chestnut, floral, and meadow honeys could be distinguished using multivariate approaches [38].

One acacia sample showed unusually high electrical conductivity compared with the other acacia samples. Because no analytical error was confirmed, this value was retained in the dataset. However, its presence should be considered when interpreting the variability of acacia conductivity. Possible explanations include a mixed floral contribution, local environmental variability, or uncertainty in declared botanical origin. Since complete melissopalynological verification was not available for all samples, botanical categories should be interpreted as declared and competition-assessed honey types rather than independently verified botanical classes.

### 4.2. Botanical Origin Versus Administrative Zone

A central question of this study was whether honey samples from the City of Zagreb differed from those from Zagreb County. The results provide limited evidence for such differences. After false discovery rate correction, none of the City–County comparisons within honey types remained statistically significant. Although flower honey showed an unadjusted difference in moisture between zones, this effect was not robust after correction for multiple testing.

The linear models confirmed this interpretation. Honey type was a significant predictor of moisture, electrical conductivity, and free acidity, whereas administrative zone was not significant for any analyzed parameter. The strongest model fit was observed for electrical conductivity and free acidity, with adjusted R^2^ values of 0.766 and 0.632, respectively. This indicates that botanical origin explained a substantial proportion of physicochemical variability, while the broad City–County classification added little explanatory power.

These findings are consistent with previous studies showing that differences among botanical types are often stronger than differences between urban and rural honey samples [11,12]. Urban ecosystems may differ from rural landscapes in vegetation structure, microclimate, and potential pollution sources, but these effects are not necessarily reflected in basic physicochemical parameters such as moisture, conductivity, acidity, and HMF. In the present dataset, the broad administrative distinction between City and County was therefore less informative than honey type.

This does not mean that urbanization has no effect on honey composition. Rather, it shows that the City–County division is too coarse to capture fine-scale environmental influences. The City of Zagreb includes parks, gardens, riparian vegetation, and suburban areas, while Zagreb County includes settlements, agricultural land, forests, and peri-urban zones. Future studies should therefore use more precise spatial descriptors, such as land-use composition around apiaries, vegetation structure, distance to roads, industrial exposure, and contaminant measurements. The present results apply to basic physicochemical quality parameters and should not be extended to environmental contamination without additional analyses. This distinction is important because honey safety assessment increasingly includes contaminants not analysed in this study, such as heavy metals, pesticide residues, veterinary drug residues, antibiotics, PAHs, PFAS, microplastics, and microbiological indicators. Therefore, the present results support conclusions on physicochemical quality, but not on overall food safety or environmental contamination status.

### 4.3. Multivariate Structure and Temporal Stability

The PCA results supported the conclusion that honey physicochemical variability was primarily structured by botanical origin. The first two principal components explained 62.8% of the total variance, while the first three components explained 83.7%. Acacia honeys were generally separated from the other types, mainly because of low electrical conductivity and low acidity. Chestnut honeys were distinguished by high conductivity, while flower and meadow honeys showed broader overlap, reflecting their more heterogeneous polyfloral composition.

The exploratory k-means results were broadly consistent with the PCA but should not be interpreted as evidence of a uniquely defined cluster structure. Internal clustering diagnostics did not identify a single optimal solution; therefore, the k = 3 solution was retained as it showed the strongest agreement with biologically expected macro-groups and provided a parsimonious summary of the main physicochemical profiles. Its value lies in summarising acacia-dominated, chestnut-dominated, and polyfloral flower/meadow-dominated groupings rather than defining a robust classification model. Similar results have been reported in chemometric studies of honey, where PCA and clustering based on physicochemical variables were useful for distinguishing botanical types [17,18,29,38].

Temporal analysis of moisture and HMF did not indicate significant long-term trends over the 2007–2019 period. Although year-to-year variation was observed, annual mean moisture values generally remained within acceptable ranges, and HMF values were consistently low. This suggests that honey quality remained relatively stable during the monitoring period. The absence of a clear increase in HMF is particularly relevant because HMF is sensitive to heating and storage conditions [37].

However, the temporal results should be interpreted cautiously. The dataset was unbalanced across years and honey types, and annual means were calculated only for year–type combinations with at least three samples. Therefore, the temporal analysis is best understood as an exploratory assessment of broad tendencies rather than a formal time-series analysis.

### 4.4. Spatial Autocorrelation and Exploratory Interpolation

Spatial analysis showed that only selected physicochemical parameters exhibited spatial structure. Global Moran’s I indicated weak but significant spatial autocorrelation for moisture and stronger spatial autocorrelation for free acidity. Electrical conductivity and HMF did not show significant global spatial autocorrelation. This suggests that some honey quality parameters may vary spatially within the Zagreb region, but this spatial signal is not uniform across variables.

Residual Moran’s I provided an important additional interpretation. After accounting for honey type, spatial autocorrelation was no longer evident for moisture, electrical conductivity, or HMF. In contrast, free acidity retained significant residual spatial autocorrelation. This indicates that the spatial pattern of free acidity was not fully explained by the geographic distribution of declared honey types. This residual spatial pattern may reflect local differences in floral composition, vegetation, microclimate, or production conditions; however, these mechanisms remain hypothetical because no direct environmental covariates were available. Therefore, the result should be interpreted as unexplained local spatial heterogeneity in free acidity, rather than as evidence of a specific environmental driver.

The interpolation results also support a cautious interpretation. IDW produced lower prediction errors than ordinary kriging for both moisture and free acidity. Kriging showed very low R^2^ values, indicating that variogram-based spatial prediction captured only a limited proportion of the observed variability. This is likely related to the moderate spatial autocorrelation, sparse and uneven sampling distribution, and limited number of observations for some parameters.

Therefore, the interpolation maps should be interpreted as exploratory visualizations of relative spatial patterns rather than as precise predictive surfaces. This aligns with previous GIS-based honey studies, where spatial mapping can provide useful insight into regional variability but requires caution when sampling density is limited [20]. In the present study, spatial methods were most informative for identifying broad tendencies and highlighting free acidity as the parameter with the clearest residual spatial structure.

The LISA analysis further confirmed that local spatial clustering of free acidity was limited. Only four of 62 samples showed significant local spatial association: two high–high clusters and two low–high outliers. Most samples were not significant. These results indicate that local spatial structure exists but is restricted to a small number of locations and should not be interpreted as evidence of widespread clustering.

### 4.5. Strengths, Limitations and Future Directions

A major strength of this study is the use of a long-term, competition-based dataset comprising honey samples collected between 2007 and 2019, analysed using standardised laboratory procedures. The study also integrates descriptive, multivariate, and spatial approaches, allowing physicochemical variability to be assessed from complementary perspectives.

Several limitations should also be considered. First, the dataset originated from a honey competition and may not represent a probabilistic sample of all honey produced in the Zagreb region. Second, botanical origin was based on beekeeper declaration and competition assessment, while complete pollen analysis was not available for all samples. Third, the City–County classification was a coarse administrative proxy and did not directly measure land use, vegetation composition, urbanisation intensity, pollution exposure, or apiary-scale environmental conditions. Fourth, the analyses focused on moisture, electrical conductivity, free acidity, and HMF, while contaminants, microbiological indicators, and other safety-related variables were not analysed. Finally, some analyses used reduced sample sizes due to missing values.

Although sensory evaluation was part of the broader competition context, sensory data were not analysed together with physicochemical and spatial variables in this study. Future research should examine whether sensory profiles correspond to the observed physicochemical grouping of honey types. Further studies should also combine physicochemical profiling with melissopalynological verification, contaminant screening, and high-resolution environmental covariates around apiaries.

## 5. Conclusions

This study analyzed the physicochemical characteristics of acacia, chestnut, flower, and meadow honeys from the Zagreb metropolitan region using a long-term Zzzagimed competition dataset collected between 2007 and 2019. Most samples met national and European honey quality requirements. Only a small proportion of samples exceeded the general moisture threshold, while none exceeded the limits for HMF or free acidity. These findings indicate that the analyzed honeys were generally of good physicochemical quality.

The results show that declared botanical origin was the main factor shaping physicochemical variability. Electrical conductivity and free acidity provided the clearest differentiation among honey types, particularly separating chestnut and acacia honeys, while flower and meadow honeys showed broader overlap consistent with their polyfloral character. Multivariate analysis further supported this pattern, as PCA and k-means clustering grouped samples primarily by honey type.

In contrast, the broad City–County classification had limited explanatory value. After correction for multiple testing, City–County differences within honey types were not statistically significant, and administrative zone was not a significant predictor in the linear models once honey type was considered. This suggests that, within the analyzed dataset, basic physicochemical parameters were influenced more strongly by botanical origin than by the administrative distinction between the City of Zagreb and Zagreb County.

Spatial analyses revealed weak to moderate spatial structure for selected parameters. Moisture showed weak global spatial autocorrelation, but this pattern did not persist after accounting for honey type. Free acidity showed the clearest spatial signal and retained significant residual spatial autocorrelation, indicating that local spatial factors may contribute to acidity variation beyond declared botanical origin. However, interpolation and LISA results should be interpreted as exploratory because spatial prediction accuracy was limited and significant local clusters were few.

Overall, the study demonstrates the value of long-term monitoring datasets for assessing the physicochemical quality and variability of honey in complex metropolitan environments.

## Figures and Tables

**Table 1:** Physicochemical parameters of Zagreb honeys by botanical type. Values are presented as available sample size, mean, standard deviation, minimum and maximum. EC, electrical conductivity; HMF, 5-hydroxymethylfurfural.

Variable		Honey Type			
		Acacia	Flower	Chestnut	Meadow
Moisture (%)	*n*	30	19	18	15
	mean	16.78	17.09	17.23	18.00
	SD	1.11	1.01	1.33	1.10
	min	15.00	14.10	15.36	15.28
	max	21.00	18.92	20.36	20.48
EC (mS/cm)	*n*	28	15	14	12
	mean	0.22	0.57	1.47	0.60
	SD	0.26	0.24	0.30	0.22
	Min	0.11	0.24	0.99	0.28
	max	1.52	1.11	1.99	1.07
HMF (mg/kg)	*n*	24	19	16	13
	mean	4.57	4.29	6.89	7.01
	SD	4.34	3.75	6.62	11.23
	min	0.02	0.00	0.38	0.00
	max	15.55	16.32	22.27	39.93
Free acidity (meq/kg)	*n*	26	12	13	11
	mean	9.77	24.54	12.91	27.38
	SD	1.85	7.66	3.64	9.55
	min	6.94	10.08	9.00	12.95
	max	13.53	37.22	21.19	42.71

*n*—number of samples, SD—standard deviation, EC—electrical conductivity, HMF—5-hydroxymethylfurfural. Values are presented as mean, standard deviation, minimum, and maximum.

**Table 2:** Linear models testing the effects of honey type and administrative zone on physicochemical parameters.

Response Variable	Model Response	Explanatory Term	F Statistics	*p*-Value	Adjusted R^2^
Moisture	Moisture	Honey type	3.824	0.013	0.087
		Administrative zone	0.205	0.652	0.087
EC	EC	Honey type	75.030	<0.001	0.766
		Administrative zone	1.448	0.233	0.766
HMF	log(HMF + 1)	Honey type	0.478	0.698	−0.037
		Administrative zone	0.033	0.856	−0.037
Free acidity	Free acidity	Honey type	36.304	<0.001	0.632
		Administrative zone	0.247	0.621	0.632

**Table 3:** Global Moran’s I statistics for physicochemical parameters.

Variable	*n*	Global Moran’s I	Global *p*-Value	Residual Moran’s I	Residual *p*-Value	Spatial Pattern
Moisture (%)	82	0.120	0.041	0.010	0.376	Weak global spatial autocorrelation; not retained after accounting for honey type
EC (mS/cm)	69	0.050	0.281	−0.059	0.736	Not significant
HMF (mg/kg)	72	−0.010	0.512	−0.058	0.737	Not significant
Free acidity (meq/kg)	62	0.209	0.002	0.266	<0.001	Significant spatial autocorrelation retained after accounting for honey type

EC—electrical conductivity, HMF—5-hydroxymethylfurfural.

**Table 4:** Cross-validated performance of spatial interpolation models for moisture and free acidity.

Variable	Model	Power/Variogram	RMSE	MAE	R^2^	*n*
Moisture	IDW	Power = 1.5	1.165	0.842	0.326	82
	Ordinary kriging	Matérn	1.550	0.941	0.013	82
Free acidity	IDW	Power = 1.0	8.961	7.223	0.061	62
	Ordinary kriging	Gaussian	9.660	7.943	0.001	62

RMSE—root mean square error, MAE—mean absolute error, *n*—number of samples for model evaluation, R^2^—coefficient of determination; IDW, inverse distance weighting; RMSE, root mean square error; MAE, mean absolute error; R^2^, coefficient of determination; *n*, number of samples used for model evaluation.

## Data Availability

The original contributions presented in this study are included in the article. Further inquiries can be directed to the corresponding author.

## Appendix A

**Table A1:** Loadings of physicochemical variables on principal components obtained through principal component analysis.

Variable	PC1	PC2	PC3	PC4
Electrical conductivity	−0.438	0.448	0.780	−0.021
Free acidity	−0.638	−0.311	−0.161	0.686
HMF	−0.212	−0.789	0.321	−0.479
Moisture	−0.597	0.284	−0.513	−0.547

**Table A2:** Percentage of variance explained by the principal components in the PCA model.

Component	Variance (%)	Cumulative (%)
PC1	34.6	34.6
PC2	28.2	62.8
PC3	20.9	83.7
PC4	16.3	100

**Table A3:** Composition of k-means clusters based on PCA scores (PC1–PC3), showing the distribution of honey types within each cluster.

Cluster	Acacia	Chestnut	Flower	Meadow	Total
1	0	1	9	9	19
2	1	10	0	0	11
3	19	0	3	0	22

**Table A4:** City–county comparisons of moisture and electrical conductivity for each honey type. *p*_adj values represent Benjamini–Hochberg false discovery rate–adjusted *p* values.

Honey Type	Variable	Test	Statistic	df	*p*-Value	*n* City	*n* County	*p*_adj
Acacia	Moisture	Mann-Whitney U test	145.500	-	0.117	12	18	0.467
	Electrical conductivity	Mann-Whitney U test	118.000	-	0.187	10	18	0.500
Chestnut	Moisture	Student *t*-test	0.422	16	0.679	6	12	0.957
	Electrical conductivity	Student *t*-test	0.491	12	0.632	5	9	0.957
Flower	Moisture	Student *t*-test	−2.820	17	0.012	5	14	0.095
	Electrical conductivity	Student *t*-test	0.055	13	0.957	5	10	0.957
Meadow	Moisture	Student *t*-test	0.296	13	0.772	6	9	0.957
	Electrical conductivity	Student *t*-test	−0.162	10	0.874	4	8	0.957

**Table A5:** Quality compliance screening for general honey quality thresholds.

Parameter	General Limit	Available Samples	Samples Exceeding Limit	Percentage Exceeding Limit (%)
Moisture	≤20%	82	3	3.7
HMF	≤40 mg/kg	72	0	0.0
Free acidity	≤50 meq/kg	62	0	0.0

**Table A6:** Sample selection flow and use of sample subsets in the analyses.

Dataset Step	Number	Use in Analysis	Reason
Full Zzzagimed database	351	Background database	All available honey samples from 2007 to 2019
Zagreb region subset	82	Descriptive and spatial analyses	Samples located within City of Zagreb or Zagreb County with coordinates
Samples with complete moisture, electrical conductivity, HMF and free acidity	52	PCA and k-means clustering	Complete-case multivariate analysis
Samples with complete free acidity	62	Moran’s I and LISA for free acidity	Variable-specific spatial analysis
Year–type combinations with sufficient replication	15	Temporal trend analysis	At least three samples per year–type combination

**Table A7:** Number of honey samples by honey type and administrative zone.

Honey Type	City	County	Total
Acacia	12	18	30
Chestnut	6	12	18
Flower	5	14	19
Meadow	6	9	15
Total	29	53	82

**Table A8:** Sensitivity summary for the unusually high electrical conductivity value in acacia honey.

Honey Type	*n* All	Mean EC, All Samples (mS/cm)	SD EC, All Samples	*n* Without Outlier	Mean EC Without Outlier (mS/cm)	SD EC Without Outlier	Maximum EC (mS/cm)
Acacia	28	0.218	0.258	27	0.169	0.041	1.519

**Table A9:** Local *Moran’s* I cluster composition for free acidity.

LISA Category	*n*
High–High	2
Low–High	2
Not significant	58
Total	62

**Table A10:** Missing data patterns by honey type and physicochemical parameter in the georeferenced Zagreb-region subset.

Honey Type	Parameter	*n* (Total)	*n* (Available)	*n* (Missing)	Missing (%)
Acacia	Electrical conductivity	30	28	2	6.7
	Free acidity	30	26	4	13.3
	HMF	30	24	6	20.0
	Moisture	30	30	0	0.0
Chestnut	Electrical conductivity	18	14	4	22.2
	Free acidity	18	13	5	27.8
	HMF	18	16	2	11.1
	Moisture	18	18	0	0.0
Flower	Electrical conductivity	19	15	4	21.1
	Free acidity	19	12	7	36.8
	HMF	19	19	0	0.0
	Moisture	19	19	0	0.0
Meadow	Electrical conductivity	15	12	3	20.0
	Free acidity	15	11	4	26.7
	HMF	15	13	2	13.3
	Moisture	15	15	0	0.0

**Table A11:** External validation of k-means solutions against biologically expected honey macro-groups. Flower and meadow honeys were combined into a single polyfloral category. Bold text represents the best value for each validation.

k	Adjusted Rand Index	Normalized Mutual Information	Purity	Macro-F1
2	0.429	0.389	0.692	0.521
3	**0.571**	**0.611**	**0.846**	**0.848**
4	0.546	0.573	**0.846**	**0.848**
5	0.468	0.465	0.827	0.833
6	0.362	0.414	0.808	0.805

## References

[1] Kiran C., Riyaz M. (2026). Understanding the Physicochemical Profile of Honey: A Comprehensive Review of Quality and Authenticity Parameters. J. Food Compos. Anal..

[2] Ministry of Agriculture Forestry and Fisheries (2025). Pravilnik o Medu; Narodne Novine 109/2025 1558.

[3] International Honey Commission Harmonised Methods of the International Honey Commission. https://ihc-platform.net/ihcmethods2009.pdf.

[4] Joint Research Centre Food Fraud: How Genuine Is Your Honey? 2023. https://joint-research-centre.ec.europa.eu/jrc-news-and-updates/food-fraud-how-genuine-your-honey-2023-03-23_en.

[5] Casula M., Corrias F., Atzei A., Milia M., Arru N., Satta A., Floris I., Pusceddu M., Angioni A. (2024). Multiresidue Methods Analysis to Detect Contamination of Selected Metals in Honey and Pesticides in Honey and Pollen. Foods.

[6] Naccari C., Ferrantelli V., Cammilleri G., Barbaccia G., Riolo P., Ferrante M.C., Procopio A., Palma E. (2025). Study of Toxic Metals and Microelements in Honey as a Tool to Support Beekeeping Production and Consumer Safety. Foods.

[7] Chirsanova A., Capcanari T., Boistean A., Siminiuc R. (2021). Physico-Chemical Profile of Four Types of Honey from the South of the Republic of Moldova. Food Nutr. Sci..

[8] Ucuncu O., Kalafat Kul M., Baltaci C., Muslu Aykoc A. (2025). Physicochemical Properties of Fifteen Flower Honey Samples from Five Districts in Türkiye. Sci. Rep..

[9] Lim A.H.R., Sam L.M., Gobilik J., Ador K., Lee Nyuk Choon J., Majampan J., Benedick S. (2022). Physicochemical Properties of Honey from Contract Beekeepers, Street Vendors and Branded Honey in Sabah, Malaysia. Trop. Life Sci. Res..

[10] Alqarni A.S., Owayss A.A., Mahmoud A.A., Hannan M.A. (2023). Physicochemical Composition of Local and Imported Honeys Associated with Quality Standards. Foods.

[11] Kavanagh S., Gunnoo J., Passos T.M., Stout J.C., White B. (2019). Physicochemical Properties and Phenolic Content of Honey from Different Floral Origins and from Rural versus Urban Landscapes. Food Chem..

[12] Quiralte D., Zarzo I., Fernandez-Zamudio M.-A., Barco H., Soriano J.M. (2023). Urban Honey: A Review of Its Physical, Chemical, and Biological Parameters That Connect It to the Environment. Sustainability.

[13] Klein A.-M., Vaissière B.E., Cane J.H., Steffan-Dewenter I., Cunningham S.A., Kremen C., Tscharntke T. (2007). Importance of Pollinators in Changing Landscapes for World Crops. Proc. R. Soc. B Biol. Sci..

[14] Potts S.G., Imperatriz-Fonseca V., Ngo H.T., Biesmeijer J.C., Breeze T.D., Dicks L.V., Garibaldi L.A., Hill R., Settele J., Vanbergen A.J. (2016). The Assessment Report on Pollinators, Pollination and Food Production: Summary for Policymakers.

[15] United Nations (2015). Transforming Our World: The 2030 Agenda for Sustainable Development. https://sdgs.un.org/2030agenda.

[16] Baldock K.C.R. (2020). Opportunities and Threats for Pollinator Conservation in Global Towns and Cities. Curr. Opin. Insect Sci..

[17] Scholz M.B.D.d.S., Quinhone Júnior A., Delamuta B.H., Nakamura J.M., Baudraz M.C., Reis M.O., Kato T., Pedrão M.R., Dias L.F., dos Santos D.T.R. (2020). Indication of the Geographical Origin of Honey Using Its Physicochemical Characteristics and Multivariate Analysis. J. Food Sci. Technol..

[18] Escuredo O., Rodríguez-Flores M.S., Míguez M., Seijo M.C. (2023). Multivariate Statistical Approach for the Discrimination of Honey Samples from Galicia (NW Spain) Using Physicochemical and Pollen Parameters. Foods.

[19] Soares S., Magalhães L., Moreira M.M., Rede D., Fernandes V.C., Viegas O., Pinto E., Almeida A., Azevedo R., Delerue-Matos C. (2024). Exploring Geographical Influences on Physicochemical Characteristics of Honey: The Montesinho Natural Park Scenario. Food Qual. Saf..

[20] Kaur P., Mishra A., Lal D. (2016). Honey Characterization Based on Physicochemical Parameters Using GIS Techniques: A Case Study in Selected States of Northern India. J. Food Process. Technol..

[21] Bonacci O., Roje-Bonacci T. (2018). Analize Indeksov Temperature Zraka Na Observatoriju Zagreb Grič v Obdobju 1881–2017. Acta Hydrotech..

[22] Radic V., Pasaric Z. (2004). Sinik Nadezda Analysis of Zagreb Climatological Data Series Using Empirically Decomposed Intrinsic Mode Functions. Geofizika.

[23] Galović L., Peh Z. (2014). Eolian Contribution to Geochemical and Mineralogical Characteristics of Some Soil Types in Medvednica Mountain, Croatia. Catena.

[24] Gajic-Capka M. (2005). Snow Regime Characteristics for Tourism in the Natural Park Medvednica, Croatia. Hrvat. Meteorološki Časopis.

[25] Seletković I., Potocic N., Ugarkovic D., Jazbec A., Pernar R., Seletković A., Benko M. (2009). Climate and Relief Properties Influence Crown Condition of Common Beech (*Fagus sylvatica* L.) on the Medvednica Massif. Period. Biol..

[26] Balakrishna Y., Manda S., Mwambi H., van Graan A. (2022). Statistical Methods for the Analysis of Food Composition Databases: A Review. Nutrients.

[27] R Core Team (2025). R: A Language and Environment for Statistical Computing. https://www.r-project.org/.

[28] (2002). Council of the European Union Council Directive 2001/110/EC of 20 December 2001 Relating to Honey. Off. J. Eur. Communities.

[29] Ma T., Zhao H., Liu C., Zhu M., Gao H., Cheng N., Cao W. (2019). Discrimination of Natural Mature Acacia Honey Based on Multi-Physicochemical Parameters Combined with Chemometric Analysis. Molecules.

[30] Moran P.A.P. (1950). Notes on Continuous Stochastic Phenomena. Biometrika.

[31] Pebesma E., Bivand R. (2023). Spatial Data Science: With Applications in R.

[32] Li L., Losser T., Yorke C., Piltner R. (2014). Fast Inverse Distance Weighting-Based Spatiotemporal Interpolation: A Web-Based Application of Interpolating Daily Fine Particulate Matter PM_2.5_ in the Contiguous U.S. Using Parallel Programming and k-d Tree. Int. J. Environ. Res. Public Health.

[33] Ohlert P.L., Bach M., Breuer L. (2023). Accuracy Assessment of Inverse Distance Weighting Interpolation of Groundwater Nitrate Concentrations in Bavaria (Germany). Environ. Sci. Pollut. Res..

[34] Shepard D. (1968). A Two-Dimensional Interpolation Function for Irregularly-Spaced Data. Proceedings of the 1968 23rd ACM National Conference.

[35] Webster R., Oliver M.A. (2007). Geostatistics for Environmental Scientists.

[36] Anselin L. (1995). Local Indicators of Spatial Association—LISA. Geogr. Anal..

[37] Shapla U.M., Solayman M., Alam N., Khalil M.I., Gan S.H. (2018). 5-Hydroxymethylfurfural (HMF) Levels in Honey and Other Food Products: Effects on Bees and Human Health. Chem. Cent. J..

[38] Šarić G., Matković D., Hruškar M., Vahčić N. (2008). Characterisation and Classification of Croatian Honey by Physicochemical Parameters. Food Technol. Biotechnol..

